# Voice signals database of ALS patients with different dysarthria severity and healthy controls

**DOI:** 10.1038/s41597-024-03597-2

**Published:** 2024-07-19

**Authors:** Raffaele Dubbioso, Myriam Spisto, Laura Verde, Valentina Virginia Iuzzolino, Gianmaria Senerchia, Elena Salvatore, Giuseppe De Pietro, Ivanoe De Falco, Giovanna Sannino

**Affiliations:** 1 Department of Neurosciences, Reproductive Sciences and Odontostomatology, University of Naples “Federico II”, Naples, 80131 Italy; 2 Department of Psychology of the University of Campania “Luigi Vanvitelli”, Caserta, 81100 Italy; 3 Department of Mathematics and Physics of the University of Campania “Luigi Vanvitelli”, Caserta, 81100 Italy; 4 Department of Advanced Biomedical Sciences, University of Naples “Federico II”, Naples, 80131 Italy; 5 Department of Information Sciences and Technologies, Pegaso University, Naples, 80143 Italy; 6 National Research Council of Italy (CNR), Institute for High-Performance Computing and Networking (ICAR), Naples, 80131 Italy

**Keywords:** Neurodegenerative diseases, Preventive medicine

## Abstract

This paper describes a new publicly-available database of VOiCe signals acquired in Amyotrophic Lateral Sclerosis (ALS) patients (VOC-ALS) and healthy controls performing different speech tasks. This dataset consists of 1224 voice signals recorded from 153 participants: 51 healthy controls (32 males and 19 females) and 102 ALS patients (65 males and 37 females) with different severity of dysarthria. Each subject’s voice was recorded using a smartphone application (Vox4Health) while performing several vocal tasks, including a sustained phonation of the vowels /a/, /e/, /i/, /o/, /u/ and /pa/, /ta/, /ka/ syllable repetition. Basic derived speech metrics such as harmonics-to-noise ratio, mean and standard deviation of fundamental frequency (F_0_), jitter and shimmer were calculated. The F_0_ standard deviation of vowels and syllables showed an excellent ability to identify people with ALS and to discriminate the different severity of dysarthria. These data represent the most comprehensive database of voice signals in ALS and form a solid basis for research on the recognition of voice impairment in ALS patients for use in clinical applications.

## Background & Summary

Amyotrophic Lateral Sclerosis (ALS) is a neurodegenerative disease characterized by a progressive loss of motor function due to the damage of motor neurons located in the motor cortex, the brain stem nuclei, and the anterior horn of the spinal cord^1^.

ALS is considered a rare disease with a low incidence and prevalence. In particular, a recent systematic review^2^ reported global incidence values ranging from 0.26 per 100,000 person-years in Ecuador to 23.46 per 100,000 person-years in Japan, while prevalence rates ranged from 1.57 per 100,000 in Iran to 11.80 per 100,000 in the United States. In Europe, overall, the average annual crude incidence ranged from 1.11 per 100,000 person-years in Serbia to 5.55 per 100,000 person-years in Denmark, while point prevalence ranged from 3.44 per 100,000 population in Malta to 10.80 per 100,000 population in Italy.

ALS causes individuals to become progressively weaker and lose motor function, eventually resulting in death. The weakness most commonly starts in the limb muscles (i.e., spinal onset), more often in distal muscles than in proximal muscles^3^.

In about 20%−30% of cases, there is a bulbar onset of the disease, presenting with dysarthria, dysphagia, and dysphonia^3^. Dysarthria is a motor disorder of speech characterized by abnormalities of the articulation and intelligibility of speech^4^, it is commonly reported as the worst part of the disease by ALS patients^5^ due to the impact of communication on overall quality of life and well-being^6,7^.

In the clinical setting, the state-of-the-art assessment of dysarthria is based on a perceptual rating scale, namely the first item of the ALS functional rating scale-revised (ALSFRS-R)^8^. Unfortunately, ALSFRS-R relies on the subjective assessment of patients’ symptoms and, therefore, lacks reliability and sensitivity to detect subclinical changes in the bulbar motor system and correctly classify patients based on clinical severity. Improved prediction accuracy of bulbar decline can be important for clinical care because decisions regarding communication intervention and palliative care are most effective when made early^9^. Early diagnosis is crucial for a better prognosis in the treatment of the disease, and, to date, no other method offers such low cost and simplicity as voice-assisted diagnosis. Hence, evaluation of speech and speaking performance may be well suited for ALS early detection and monitoring.

In this context, several automated speech analysis methods have been used in the last years to add value to ALS diagnosis by detecting subclinical changes. These studies have applied different machine learning approaches, in some cases achieving high performance in terms of diagnostic accuracy using simple tasks such as the sustained phonation of vowels or syllable repetition^10–16^. For example, by applying an interpretable decision tree model, our group found that a simple task such as vocalization was able to distinguish healthy controls from ALS patients, with higher accuracy performance in patients with more severe dysarthria^16^.

However, no study has made raw voice signals publicly available by using different tasks, nor has it recruited clinically well-characterized patients, including patients with different severity of dysarthria, and has not included, in most cases, age- and sex-matched healthy controls with the ALS population. For instance, as far as we know, only one study^15^ has made vocal signals public, but including only one task (i.e., phonation of the vowel /a/), and was performed on a sample of 15 patients affected by ALS not matched for age and sex with the healthy control group. A subsequent study^10^, instead, did not share their data publicly but only by request of qualified investigators and analysed a relatively large sample of 67 ALS patients, but without specifying the severity of dysarthria and including only one task such as the description of a picture.

Based on this background, VOiCe signals database of ALS patients (VOC-ALS)^17^ represents the most comprehensive and freely downloadable dataset of vowel phonations and syllables repetition recorded in healthy controls and ALS patients with different dysarthria severity, recruited consecutively during routine outpatient visits at the ALS center of the Federico II University Hospital of Naples, Italy. This dataset^17^ consists of 1224 voice samples from 153 participants, 102 ALS patients, and 51 age- and sex-matched healthy controls. Besides voice samples, VOC-ALS^17^ makes available demographic, history and clinical information of participants and basic acoustic features extracted from the voice samples. All together the data provided for each patient could potentially offer additional insight into how specific disease features are associated with speech metrics.

We believe that a better understanding of speech abnormalities in ALS patients could contribute to the development of technology to augment communicative interactions and help answer critical questions around the emergence of language and communication deficits across all stages of the disease in order to detect sensitive biomarkers for an early diagnosis and a proper prognosis. Finally, we hope that the published dataset will engage other researchers in this critical field of study.

## Methods

### Experimental Study

**Fig. 1:**
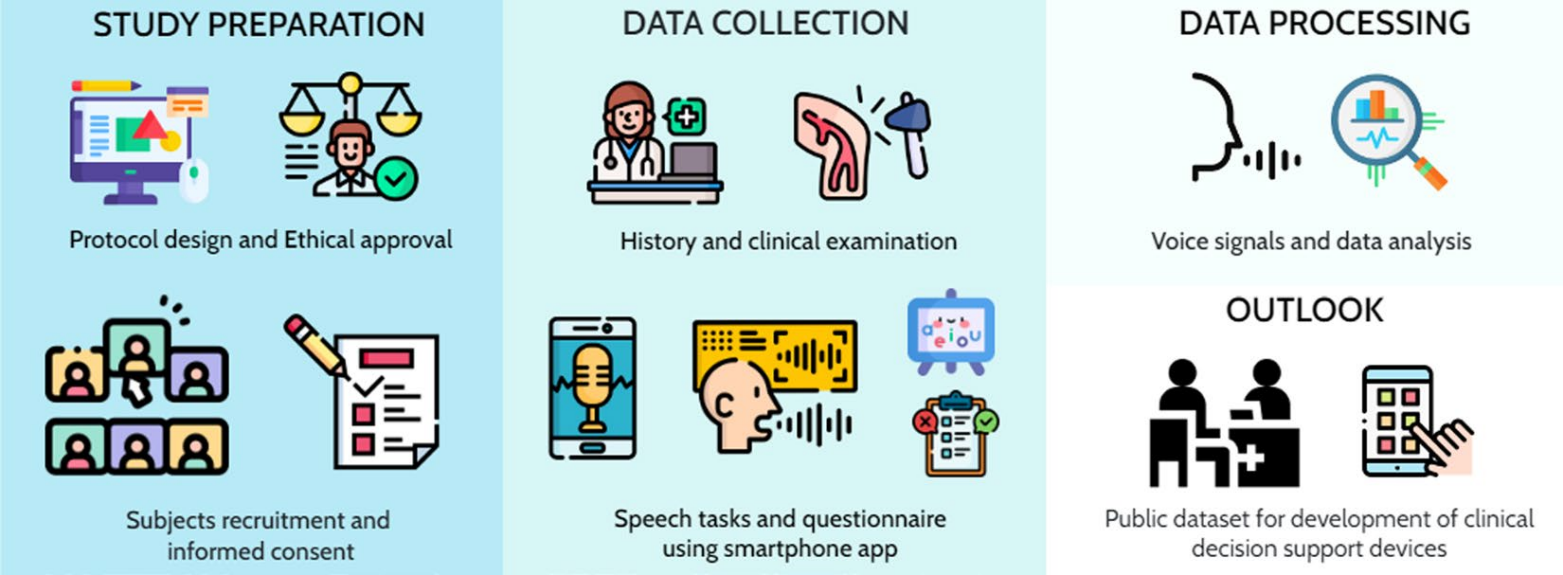
VOC-ALS: schematic overview of the experimental study for the dataset creation.

The research study consisted of three phases, summarized in Fig. 1: (i) the Study Preparation, (ii) the Data Collection, and (iii) the Data Processing, each of which was characterized by different steps. The figure also shows the outlook of the study, namely a new and freely downloadable dataset that will be used by the scientific community for any appropriate purposes.

#### Study Preparation

The first step of the *Study Preparation* phase was the **Protocol Design** with its **Ethical Approval**. In fact, data collection followed an appropriate protocol based on requirements provided by Standard Protocol Items: Recommendations for Interventional Trials (SPIRIT) 2013 standard^18^. The SPIRIT guidelines consist of a list of recommended items to be included in a clinical trial protocol to enhance its transparency and completeness. The checklist covers the essential information that should be present in a well-designed and well-conducted clinical trial protocol.

The protocol designed for this study was composed of an *Administrative Information* section which contains all relevant administrative details and necessary information; a *Methods* section which provides a comprehensive description of the procedures used to set up the database; an *Ethics and Dissemination* section, where the plans for obtaining ethic approval and the dissemination strategy are outlined; and, finally, the *Appendix* which contains all supporting documents, including consent forms and medical history forms.

The entire protocol was reviewed and approved by the medical research Ethics Committee of “Federico II" University Hospital of Naples (Italy), with trial numbers *Protocol ID: 100/17/ES01* and *93/2023*. The research was carried out in accordance with the Declaration of Helsinki and the participants provided their written informed consent before inclusion in the study.

The second step of the *Study Preparation* phase was characterized by the **Recruitment of Subjects** and the **Collection of Informed Consent** from each of them. An adequate recruitment phase was ensured and involved a sufficient number of participants referring to the ALS centre of the University Hospital Federico II of Naples between 01/01/2022 and 09/30/2023, including healthy controls (i.e., caregivers) and people with ALS. During the recruitment phase, participants were informed about the study’s objectives. Those who expressed interest in participating in the study were provided with the necessary bureaucratic documents, including the information sheet and informed consent forms. Therefore, the invitation to participate in the study was extended to individuals who met the specified inclusion and exclusion criteria.

Subjects considered eligible for the study met the following inclusion criteria:

- Italian-speaking subjects aged between 18 and 90;
- subjects able to comply with the study visit schedule and other protocol requirements;
- healthy controls and people with ALS fulfilling the revised El Escorial criteria^19^;

Subjects with the following exclusion criteria were, instead, excluded:

- subjects younger than 18 years or older than 90 years;
- subjects with illnesses such as colds or upper respiratory tract infections;
- subjects with other neurological disorders that may affect voice or speech (e.g. Alzheimer’s disease, Parkinson’s disease, stroke);
- patients that scored zero at the first item of ALSFRS-R, namely patients with loss of useful speech.

Only participants who met the inclusion criteria and who had given informed consent were enrolled and registered in the study by assigning them an identification code for the pseudonymization of their data.

#### Data Collection

Registered participants had access to the **Clinical Assessment**, which is the first step of the study’s second phase, namely the *Data Collection* phase. During this step, each participant underwent a detailed clinical evaluation, which included collecting demographic and medical history information and, for ALS patients, some medical tests to assess the severity of the disease.

Demographic data such as gender and date of birth for each participant was recorded, and, only for healthy controls, we asked for medical history data such as the presence of any diseases potentially affecting speech.

Additionally, we recruited ALS patients meeting the revised El Escorial criteria for the diagnostic categories “possible”, “probable,” “probable laboratory supported,” or “definite”^19^ and recorded the diagnostic delay determined as the time from patient-reported first symptom onset to formal diagnosis by a physician and disease duration, computed as time from symptom onset to testing. Patients’ disease severity was assessed by dedicated clinical scales and instruments:

- the manual muscle testing scale established by the Medical Research Council of the Royal College of Physicians and Surgeons (MRC scale)^20^ to evaluate muscle strength in each body region. Specifically, each patient was evaluated by the same trained neurologist (R.D.). Inside the clinic and in a supine or sitting position, the various muscles were evaluated, starting from the head region, then the muscles of the upper limbs and finally those of the lower limbs. Depending on the degree of muscle impairment, the patient was asked to perform a movement against resistance, against gravity or in zero gravity. The MRC scale^20^ scores each tested muscle from 0 (paralysis) to 5 (normal strength). The neurologist (R.D.) examined three muscles in the head region: score 0 to 15; seven muscles for each side of the upper limbs: score 0 to 70 points; and six muscles for each side of the lower limb: score 0 to 60 points^21^. The muscles tested for the head region were the neck flexor, sternocleidomastoid, and orbicularis oris. Regarding the upper limbs, we evaluated the deltoid, biceps and triceps brachii, wrist and finger extensor, abductor pollicis brevis and first dorsal interosseous muscles. Lastly, for lower limbs, we assessed iliopsoas, quadriceps, biceps femoris, tibialis anterior, gastrocnemius and extensor digitorum brevis muscles.
- the Penn Upper Motor Neuron Score (PUMNS)^22^ was used to assess the Upper Motor Neuron (UMN) burden. The same trained neurologist (R.D.), by using a reflex hammer or considering specific neurological signs, evaluated the UMN impairment by applying this well-recognized and standardized scale. PUMNS scale ranges from 0 (normal) to a maximum of 32 (for widespread/severe UMN involvement) and evaluates the bulbar region (scores 0 to 4), upper limbs (scores 0 to 14) and lower limb (scores 0 to 14). Specifically, regarding the bulbar region: one point each is assigned for a present jaw jerk, facial reflex, palmomental sign, and score ≥13 on the Central Nervous System (CNS)-Lability Scale^23^. The CNS-Lability Scale is a validated, self-administered, 7-item questionnaire that provides a quantitative measure of pseudobulbar affect symptoms. For upper and lower limbs, a single point is given for a pathologically brisk reflex (triceps, biceps, finger flexors, patellar, crossed adduction, Achilles), presence of clonus, and presence of Hoffman’s and Babinski signs. Additionally, one or two points may be assigned for increased scores on the modified Ashworth Spasticity Scale^24^ for each limb.
- The ALSFRS-R^8^ includes 12 questions that can have a score of 0 to 4. A score of 0 on a question would indicate no function, while a score of 4 would indicate full function; thus, impairment of a specific function was considered if patients scored less than 4 on the target question. Questions 1 to 3 are related to bulbar function (speech, salivation and swallowing), questions 4 to 9 are related to limb function and questions 10-12 are related to respiratory function. The degree of severity of dysarthria was assessed for each patient based on the first item of the ALSFRS-R scale. Namely, the non-dysarthric patient corresponded to a normal speech process (score 4), the patient with mild dysarthria indicated detectable speech disturbances (score 3), the moderate dysarthric patient corresponded to intelligible speech with repetitions (score 2), the patient with severe dysarthria indicated language combined with non-vocal communication (score 1). The ALSFRS-R was also used to compute the disease progression rate by applying the following formula^25^: ΔALSFRS-R = (48 - ALSFRS-R at the study inclusion)/(disease duration in months). Finally, the ALSFRS-R scale was administered by an experienced neurologist (R.D.) via in-person interview.
- The King’s clinical stage is closely linked to the anatomical spread of the disease^26^. The King’s staging system consists of five disease stages: 1= one region involved; 2= two regions involved; 3= three regions involved; 4A= patient needs gastrostomy; 4B= patient needs non-invasive ventilation^27^. The stage can be derived from direct observation of the patients and from the ALSFRS-R scale^26^.
- Respiratory function was also assessed through a portable spirometer (Pony Fx, COSMED S.R.L, Italy). The spirometry was performed with the patient sitting upright. Results for forced vital capacity (FVC) were expressed as a percentage of predicted value from an average of three trials^28–30^.

Lastly, we performed the genetic analysis in all patients by using a clinical panel test including the four most common genes, namely *C9orf72*, *SOD1*, *TARDBP* and *FUS*^31^.

The second step of the *Data Collection* phase was the **Voice Data Acquisition**, during which the registered participants performed the speech tasks and filled out a self-report questionnaire on dysarthria^32^.

Speech recordings were carried out during outpatient visits at the ALS center of the Federico II University Hospital of Naples. The outpatient room was quiet with a low ambient noise level ( < 30 dB of background noise) and dryness (humidity rate above 35−40%), and in the absence of emotional and physiological stress for the participants. Voice recordings were acquired by using a dedicated m-health system, Vox4Health^33,34^, able to record in real time the voice signal by using the microphone of a mobile device. This system was installed on a Samsung Galaxy S8+ SM-G955F with Android version 9.0 operating system. The microphone of the mobile device, i.e. the smartphone, was held at a distance of about 20 cm from the patient at an angle of about 45 degrees.

We recorded each subject during a single session in the presence of a trained investigator (M.S.), and all participants performed the following five speech tasks:

- Sustained vowel production: recording vocalizations of each vowel (/a/, /e/, /i/, /o/, and /u/) for a minimum of 5 seconds each, ensuring a continuous loudness^32,35^;
- diadochokinetic task: recording of the patient’s voice while repeating the syllables /pa/, /ta/, /ka/ as fast as possible in a single breath in three different audio files^32,35^;
- automatic speech task: recording the patient vocalizing the days of the week in Italian language in consecutive order, starting from Monday^35^;
- reading a text: recording the patient during the reading of a short text in the Italian language entitled “Il vento del Nord”^35^ ("The North Wind" in English);
- monologue: recording of the patient’s voice while describing, for at least 60 seconds, a picture in which the members of a family are depicted in the foreground, from whose expressions one perceives a serene and happy soul; in the background of the cartoon, two thieves are depicted in the act of stealing^36^.

All recordings were sampled at 8000 Hz with a 16-bit resolution and saved in *wav* format.

Lastly, all participants completed a self-report questionnaire on dysarthria^32^. This self-assessment questionnaire consisted of 35 items concerning four main domains: perceived characteristics of the disorder, situational difficulty, compensatory strategies used, and perceived reactions of others. The subject was asked to agree or disagree with each statement, using a five-point scale ranging from 0 ("never”) to 4 ("always”), thus the score ranged from 0 to 140. More details about the questionnaire and its scores are reported in section **Participants’ Clinical and Acoustic Data**.

**Fig. 2:**
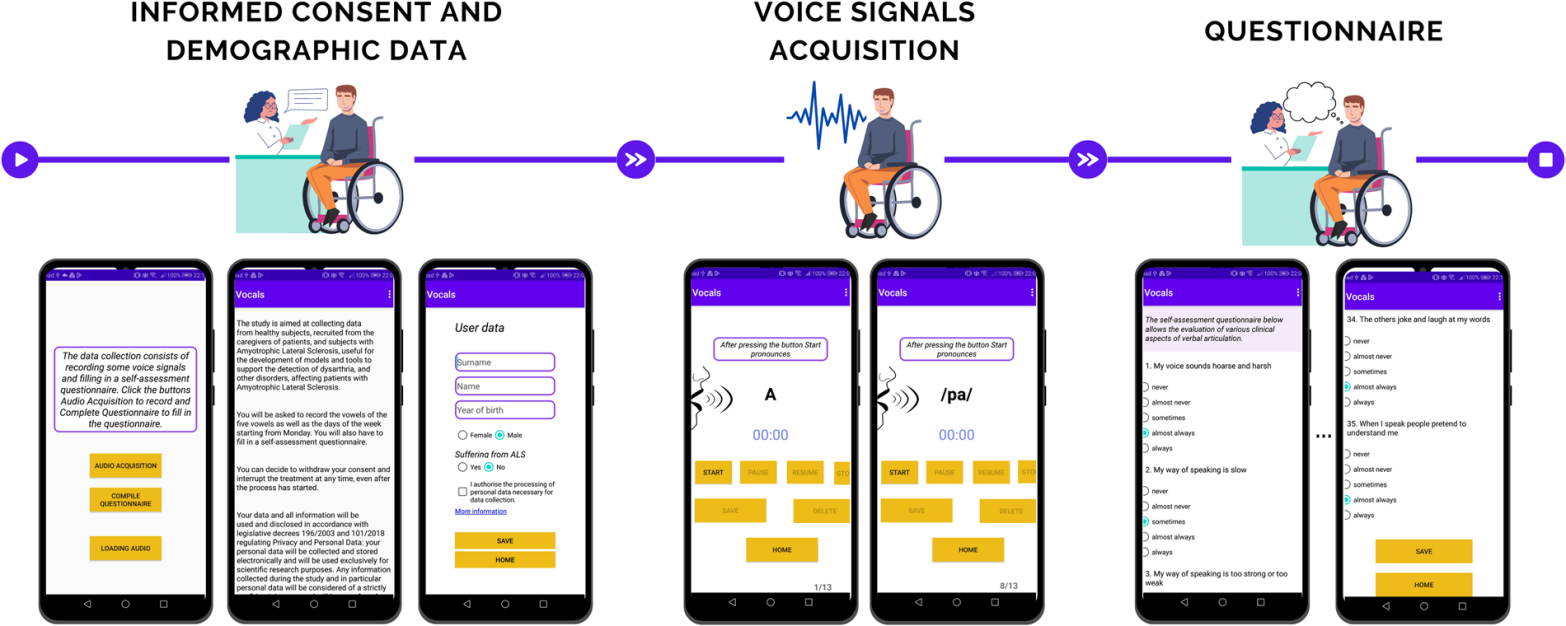
Vox4Health smartphone application provided to participants.

As with the speech tasks, the questionnaire was also collected using Vox4Health^34^. An overview of this app is detailed in Fig. 2.

#### Data Processing

Finally, the study concluded with the *Data Processing* phase, during which the voice signals were processed by extracting a set of features, and all data were analyzed.

For each voice signal, multiple acoustic features were computed using the Parselmouth^37^, a Python library for the Praat software^38^. We developed a Python script to calculate the Fundamental Frequency (F_0_) mean, the standard deviation of F_0_, jitter, shimmer, and Harmonic to Noise Ratio (HNR).

The F_0_, also called pitch, of a speech signal refers to the approximate frequency of the (quasi-)periodic structure of the voice signals. In the Praat library, the pitch is calculated using an algorithm based on the autocorrelation method^39^. In our study, we computed the mean and the standard deviation of the F_0_.

Jitter (%) was defined as cycle-to-cycle and short-term perturbation in the fundamental frequency of the voice. In this study, we considered the local Jitter, representing the average absolute difference between two consecutive periods divided by the average period.

The shimmer (%) was defined as cycle-to-cycle and short-term perturbation in the voice amplitude. As in the case of Jitter, in this study, we refer to the local Shimmer as the average absolute difference between the amplitudes of consecutive periods divided by the average amplitude.

Finally, the HNR was obtained as the ratio of the energy of a periodic signal to the energy of the noise in the signal, expressed in dB. It is affected by both the shimmer and jitter and is referred to as the average ratio of harmonics to non-harmonics^40^.

## Data Records

As mentioned above, we enrolled 153 participants, 97 males and 56 females, with a prevalence of male participants, due to the higher incidence of the ALS in the male population^41^. Therefore, we kept a very similar M:F ratio for healthy controls as well. Indeed, the ALS group consisted of 65 males and 37 females, and the group of healthy controls of 32 males and 19 females. Table 1 details the number of participants, distinguishing between healthy and ALS subjects, and among patients, the different classes depending on the severity of the dysarthria. Specifically, we have indicated the number and percentage of female and male subjects with the mean age for each dysarthria category.

**Table 1:** Demographic data of healthy controls and patients, classified according to the severity of dysarthria, recruited in the VOC-ALS dataset^17^.

Class	Female (%)	Male (%)	Total (%)	Age (average)	
				Female	Male
*Severe-Dysarthric*	3 (2.0%)	2 (1.3%)	5 (3.3%)	65.67	64.00
*Moderate-Dysarthric*	4 (2.6%)	9 (5.9%)	13 (8.5%)	72.00	65.44
*Mild-Dysarthric*	10 (6.5%)	21 (13.7%)	31 (20.3%)	60.90	65.48
*Non-Dysarthric*	20 (13.1%)	33 (21.6%)	53 (34.6%)	61.55	60.15
**ALS patients**	**37 (24.2%)**	**65 (42.5%)**	**102 (66.7%)**		
*Healthy controls*	19 (12.4%)	32 (20.9%)	51 (33.3%)	65.00	61.78
**Total (ALS + HC)**	**56 (36.6%)**	**97 (63.4%)**	**153 (100%)**		

Demographic data of patients belonging to each class of dysarthria severity and healthy controls. Gender is expressed as number and percentage, and age as mean expressed in years for each gender category. Severe-dysarthric= patients scoring 1 at the first item of the ALSFRS-R; Moderate-dysarthric= patients scoring 2 at the first item of the ALSFRS-R; Mild-dysarthric= patients scoring 3 at the first item of the ALSFRS-R; Non-dysarthric= patients scoring 4 at the first item of the ALSFRS-R.

**Table 2:** Number of participants in the studies on ALS found in the literature (in chronological order).

Reference	Year	ALS	Control	Total
^15^	2019	15	39	54
^14^	2019	25	25	50
^10^	2020	67	33	100
^13^	2021	31	33	64
^11^	2021	45	18	63
^12^	2023	45	18	63
^16^	2024	74	23	97
**This paper**	2024	102	51	153

It should be remarked here that the number of ALS patients enrolled for this study is larger than those reported in the other papers in the literature, as Table 2 shows. In it, for each paper, the publication year is reported together with the numbers of people with ALS, healthy controls, and the total number of participants.

VOC-ALS data^17^ is made available on the Synapse platform at the link 10.7303/syn53009474. To access the data, researchers must be registered as Synapse users with a *Registered User* profile, following the instructions at https://help.synapse.org/docs/Synapse-User-Account-Types.2007072795.html. All Synapse users are required to abide by the principles and policies described in the Synapse Governance and the Synapse Terms of Use. On the Synapse platform, by selecting the VOC-ALS tab, users can access the wiki page that describes the database and how to access the data. Specifically, to access the data, users must select the *Files* tab and select the desired data folder or files. More details regarding the organization and the content of each folder and file contained in the database^17^ are reported in the following subsections.

### Voice Recordings

For privacy reasons, we could make available only the signals regarding the first two voice tasks planned in the **Voice Data Acquisition** step of the *Data Collection* phase, i.e., the prolonged phonation of vowels and the diadochokinetic task. In fact, the automatic vocal task, the reading of a text and the monologue could reveal the identity of the speaker. More information about this decision is reported in section **Participants’ Anonymity Protection**. Thus, from here on, we will only refer to the raw data and analyses derived from vowel productions and syllable repetition. Therefore, in the VOC-ALS database^17^, we have a total of 1224 voice recordings saved in *wav* format: eight live speech recordings for each subject - five recordings related to vocalization and three to the repetition of syllables.

The voice recordings have been organized into eight folders, each containing the different voice tasks performed by participants.

Here is the label of each folder:

- *phonationA:* contains vocalizations of the vowel /a/;
- *phonationE:* contains vocalizations of the vowel /e/;
- *phonationI:* contains vocalizations of the vowel /i/;
- *phonationO:* contains vocalizations of the vowel /o/;
- *phonationU:* contains vocalizations of the vowel /u/;
- *rhythmPA:* contains repetitions of the syllable /pa/;
- *rhythmTA:* contains repetitions of the syllable /ta/; and
- *rhythmKA:* contains repetitions of the syllable /ka/.

Each file is named in each folder with the corresponding participant ID and the signal label; for example, *PZ001_phonationA* indicates the vocalization of vowel /a/ of the patient with ID number PZ001.

### Participants’ Clinical and Acoustic Data

As an accompaniment of the voice records in *wav* format, a *.xlsx* file is released in VOC-ALS^17^. This file contains all information collected during the study. In detail, it reports the list of the recruited subjects, each of them identified with their unique ID, the clinical and acoustic features, and the score related to the self-report questionnaire on dysarthria.

More specifically, each row of the .xlsx file is composed of:

1. **ID**: a combination of letters and numbers generated to identify each subject uniquely and for the pseudonymization of the data;
2. **age**: expressed in years;
3. **sex**: *M* for males and *F* for females;
4. **category**: *HC* for Healthy Controls; *ALS* for Amyotrophic Lateral Sclerosis patients;
5. **clinical features**: basic and specific clinical scales performed during the **Clinical Assessment** of ALS patients. Clinical data of each patient are available in the .xlsx file released as an integration of the VOC-ALS dataset^17^.
  These include:
  the onset region of the disease (OnsetRegion): spinal or bulbar;
  therapy (Therapy) was defined as the disease-modifying treatment at the time of testing;
  genetic analysis (GeneticTest): negative or positive for mutations in the following genes, such as *C9orf72*, *SOD1*, *TARDBP* and *FUS*;
  diagnostic delay (DiagnosticDelay) meant the time from patient-reported first symptom onset to formal diagnosis by a physician;
  disease duration (DiseaseDuration) is meant as the time from symptom onset to testing;
  forced vital capacity (FVC%) expressed as a percentage of predicted value from an average of three trials;
  the ALSFRS-R total score (ALSFRS-R_TotalScore) ranging from 0 to 48;
  the disease progression rate (ProgressionRate) expressed as the ALSFRS-R points lost/month;
  the Revised El-Escorial Criteria (Revised_ElEscorial_Criteria) including the category of definite, probable, probable laboratory-supported, or possible ALS;
  the ALSFRS-R speech subscore (ALSFRS-R_SpeechSubscore) ranging from 0 to 4, where 0 corresponds to loss of useful speech, 1 to speech combined with nonvocal communication, 2 to intelligible with repeating, 3 to detectable speech disturbance and 4 to normal speech process. This subscore was also used to classify the severity of dysarthria in our patients’ cohort. We, therefore, defined four classes: severe-dysarthric patients with a score of 1; Moderate-dysarthric patients with a score of 2; Mild-dysarthric patients with a score of 3; and Non-dysarthric patients with a score of 4;
  the ALSFRS-R salivation subscore (ALSFRS-R_SalivationSubscore) ranging from 0 to 4, where 0 corresponds to marked drooling (requires constant tissue or handkerchief) and 4 to normal function;
  the ALSFRS-R swallowing subscore (ALSFRS-R_SwallowingSubscore) ranging from 0 to 4, where 0 corresponds to nothing by mouth (exclusively parenteral or enteral feeding) and 4 to normal function;
  the ALSFRS-R hand-writing subscore (ALSFRS-R_HandwritingSubscore) ranging from 0 to 4, where 0 corresponds to unable to grip pen and 4 to normal function;
  the ALSFRS-R cutting food and handling utensils subscore (ALSFRS-R_CuttingFoodSubscore) ranging from 0 to 4, where 0 corresponds to needs to be fed and 4 normal function;
  the ALSFRS-R cutting food and handling utensils with gastrostomy subscore (ALSFRS-R_CuttingFoodWithGastro stomySubscore) ranging from 0 to 4, where 0 corresponds to unable to perform any aspect of task and 4 normal function;
  the ALSFRS-R dressing and hygiene subscore (ALSFRS-R_DressingHygieneSubscore) ranging from 0 to 4, where 0 corresponds to total dependence and 4 to normal function;
  the ALSFRS-R turning in bed and adjusting bed clothes subscore (ALSFRS-R_TurningBedSubscore) ranging from 0 to 4, where 0 corresponds to helpless and 4 normal function;
  the ALSFRS-R walking subscore (ALSFRS-R_WalkingSubscore) ranging from 0 to 4, where 0 corresponds to no purposeful leg movement and 4 to normal function;
  the ALSFRS-R climbing stairs subscore (ALSFRS-R_ClimbingStairsSubscore) ranging from 0 to 4, where 0 corresponds to cannot do and 4 to normal function;
  the ALSFRS-R dyspnea subscore (ALSFRS-R_DyspneaSubscore) ranging from 0 to 4, where 0 corresponds to significant difficulty, considering using mechanical respiratory support and 4 to normal function;
  the ALSFRS-R orthopnea subscore (ALSFRS-R_OrthopneaSubscore) ranging from 0 to 4, where 0 corresponds to unable to sleep and 4 to normal function;
  the ALSFRS-R respiratory insufficiency subscore (ALSFRS-R_BreathingInsufficiencySubscore) ranging from 0 to 4, where 0 corresponds to invasive mechanical ventilation by intubation or tracheostomy and 4 to normal function;
  the King’s clinical stage (KingClinicalStage) ranging from 1 to 4A/B, where 1 corresponds to the first region involved, and 4A to nutritional failure, 4B to respiratory failure;
  MRC for head muscles (MRC_HeadMuscles) ranging from 0 to 15, where 0 corresponds to paralysis and 15 normal strength in all examined muscles;
  MRC for upper limb muscles (MRC_UpperLimbMuscles) ranging from 0 to 70 where 0 corresponds to paralysis and 70 normal strength in all examined muscles;
  MRC for lower limb muscles (MRC_LowerLimbsMuscles) ranging from 0 to 60, where 0 corresponds to paralysis and 60 normal strength in all examined muscles;
  the PUMNS bulbar subscore (PUMNS_BulbarSubscore) ranging from 0 to 4, where 0 corresponds to normal and 4 widespread/severe upper motor neuron involvement in the examined district;
  the PUMNS upper limbs subscore (PUMNS_UpperLimbsSubscore) ranging from 0 to 14, where 0 corresponds to normal and 14 widespread/severe upper motor neuron involvement in the examined district;
  the PUMNS lower limbs subscore (PUMNS_LowerLimbsSubscore) ranging from 0 to 14, where 0 corresponds to normal and 14 widespread/severe upper motor neuron involvement in the examined district;
6. **acoustic features**: measurements of the acoustic features calculated for each .wav file, as described in the **Data Processing** phase, that is the F_0_ mean (meanF0), the standard deviation of F_0_ (stdevF0), the harmonics-to-noise ratio (HNR), jitter (localJitter), and shimmer (localShimmer);
7. **questionnaire score**: total score obtained from the sum of the 35 items of the self-assessment questionnaire^32^ (Cantagallo_Questionnaire). This self-assessment questionnaire is the Italian version of the Yorkston, Bombardier and Hammen questionnaire^42^ translated into Italian by Schindler and Gullì^43^. This questionnaire is designed to assess the individual experience of a subject with dysarthria and to identify areas where additional support may be needed. In particular, 35 questions are asked about speech characteristics (e.g., hoarseness, speech rate, volume too high or too low), difficulties in communicating with others, both by talking on the phone and in person, and in particular environmental contexts. The strategies used to compensate for communication difficulties are also explored, and the reactions of others to the speech of the patient with dysarthria are assessed. The patient is asked to answer each question by choosing from five options: “*never*", “*almost never*", “*sometimes*", “*almost always*", and “*always*". Each of these options is given a score from 0 (*never*) to 4 (*always*). The final score is the sum of the scores obtained for each question. The final score thus obtained can be interpreted as follows:
  0-6: dysarthria absent or not perceived;
  7-34: dysarthria perceived as mild impairment;
  35-69: dysarthria perceived as moderately disabling;
  70-133: dysarthria perceived as severe disability;
  134-140: dysarthria perceived as total disability.

### Participants’ Anonymity Protection

In this study, we used pseudonymization, a technique defined by the General Data Protection Regulation -GDPR-^44^ and the Data Protection Act 2018^45^. Pseudonymization processes personal data so it can’t be linked to an individual without additional information. Specifically, we replaced the names of participants with unique IDs. Only the medical staff who registered and clinically evaluated the subjects have the link between pseudonymized and raw data, stored locally at the ALS center of the “Federico II" University Hospital of Naples, Italy.

To ensure anonymity, the VOC-ALS database^17^ on Synapse only includes signals from two voice tasks: the prolonged vowel phonation and the diadochokinetic task. Voice recordings (combined, if necessary, with other elements) can reveal identity and are protected as personal data under GDPR^44^, potentially qualifying as a special category of data under Article 9^44^. This article restricts the processing and circulation of such data when certain conditions are met.

It should be remarked here that recordings of reading, monologue, and days of the week are highly identifiable and contain clear voice and speech. The monologue, where the patient describes a picture for at least 60 seconds, could reveal private information, increasing the identifiability risk. Thus, these recordings cannot be disclosed.

Additionally, voice counterfeiting is a significant issue, highlighted in the Guarantor’s Vademecum on Deepfakes, which states that AI can realistically recreate voices from real content.

## Technical Validation

### Clinical Analysis

For this section, we refer to the data contained in the columns from 2 to 21 of the .xlsx file. History and clinical examination showed no abnormalities in healthy controls. Regarding subjects with ALS, the percentage of patients showing dysarthria was similar to those without dysarthria; furthermore, the former group included older participants and a more severe disease compared to patients without dysarthria (Table 3). Specifically, dysarthric patients showed lower scores on FVC, ALSFRS-R, bulbar subscore, a faster rate of disease progression and a shorter diagnostic delay, and a higher proportion of patients belonging to late clinical disease stages according to King’s classification. Considering the whole ALS group, among the bulbar symptoms (i.e., speech, salivation or swallowing impairment), the most experienced complaint was speech abnormality in 48% of patients (49/102), followed by swallowing disorders in 44% (45/102) and salivation problems in 36% (37/102). Considering the subgroup of patients with dysarthria, eating problems or excessive salivation were present in 76% (37/49) and in 61% (30/49) of patients, respectively, while these symptoms were rare in the subgroup without dysarthria (Table 3).

**Table 3:** Demographic and clinical data of ALS patients classified according to the presence of the dysarthria symptom.

	ALS-all	ALS without dysarthria	ALS with dysarthria
**No**.	102	53 (52%)	49 (48%)
**Male, n (%)**	65 (64%)	33 (62%)	32 (65%)
**Age at testing, y, median (IQR)**	64 (15)	62 (13)	68 (17)
**Bulbar onset, n (%)**	22 (20%)	0 (0%)	20 (41%)
**Disease duration, m, median (IQR)**	15.5 (29.75)	22 (33)	12 (23)
**Diagnostic delay, m, median (IQR)**	10.5(16)	14(19)	9(10)
**FVC% of predicted, median (IQR)**	85 (40.25)	92 (19)	74 (47)
**ALSFRS-R points lost/month (progression rate), median (IQR)**	0.73 (0.95)	0.44 (0.56)	1.2 (1.5)
**ALSFRS-R total score (0-48), median (IQR), higher is better**	36 (12)	39 (12)	33 (12)
**Bulbar subscore (0-12), median (IQR), higher is better**	11 (3.75)	12 (0)	8 (3)
**Speech subscore (0-4), median (IQR), higher is better**	4 (1)	4 (0)	3 (1)
Excessive salivation at ALSFRS-R, n (%)*	37 (36%)	7 (13%)	30 (61%)
Swallowing impairment at ALSFRS-R, n (%)*	45 (44%)	8 (15%)	37 (76%)
Dysarthria severity % (1,2,3,4)**, higher is better	5%, 13%, 30%, 52%	0%, 0%, 0%, 100%	10%, 27%, 63%, 0%
King’s stage % (1,2,3,4)***, lower is better	28%, 26%, 31%, 14%	45%, 32%, 13%, 9%	10%, 20%, 51%, 18%
UMN bulbar subscore (0-4)****, median (IQR), lower is better	1 (2)	1 (1)	2 (2)
**Genetic testing, positive (%)**	6 (5.9%)	3 (5.7%)	3 (6.1%)
**Self-report questionnaire on dysarthria (0-140), median (IQR), lower is better**	20 (47.5)	12 (17)	50 (53.5)

ALS patients without dysarthria are patients scoring 4 at item 1 (speech) of the ALSFRS-R scale; ALS patients with dysarthria are patients scoring <4 at item 1 (speech) of the ALSFRS-R scale.

*patients that scored <4 at item 2 (salivation), item 3 (swallowing) of the ALSFRS-R scale.

**percentage of patients belonging to each score at item 1 of the ALSFRS-R scale.

***percentage of patients belonging to each stage of King’s scale.

****calculated with The Penn Upper Motor Neuron Score.

IQR = interquartile range; ALSFRS-R = revised amyotrophic lateral sclerosis functional rating scale; UMN = upper motor neuron; FVC = forced vital capacity.

Lastly, genetic analysis was negative in all patients but six, four patients harbouring the hexanucleotide repeat expansion of the *C9orf72* gene (3.9%), one with a SOD1 gene mutation (1%) and one with a *TARDBP* gene mutation (1%).

Demographic and clinical data of the study populations are shown in detail in Table 3.

### Acoustic Analysis

In this section, we make reference to the 40 columns of the dataset containing the numerical values of the 40 acoustic features calculated over the .wav file of the vowels and syllables, i.e., the columns from 22 to 61. The limitation of referring to only these features is because, as already mentioned before, for the participants’ anonymity protection, we could make available only the signals, i.e. the .wav files, regarding the first two voice tasks planned in the **Voice Data Acquisition** step of the *Data Collection* phase, i.e., the prolonged phonation of vowels and the diadochokinetic task.

Whenever a new dataset is introduced in the literature, an investigation of the data is important to examine its quality, evaluate its features, and inquire into the possibility of suitably working with it. To fulfil this issue, we have performed many such tests by using Matlab^46^ and Python^47^.

Reporting the results from all of these tests would require too much space; therefore, here we summarize the main results achieved.

- *is parametric statistical analysis viable?*: this analysis, relying on parametric tests as, e.g., one-way analysis of variance (ANOVA)^48^ and t-test analysis^49^, can only be performed if the three hypotheses of *normality*, *homoscedasticity*, and *independence* are satisfied in the dataset. We have checked them as reported below.
  - *normality*: we have used the Shapiro-Wilk test^50^ over each dataset parameter to investigate whether or not its values come from a normal distribution. The result is that, at a significance level *α* = 0.05, for the vast majority of the parameters, their values do not come from a normal distribution. The exceptions to this general behaviour are represented by the six parameters representing the *HNR* for the vowels /a/, /e/, and /i/ as well as for the syllables /pa/, /ta/, and /ka/.
  - *homoscedasticity*: we have availed ourselves of the Bartlett test^51^ to check if, for any considered parameter, the population variances of the different classes are equal or if there are at least two such variances that are different. The result is that, for 18 out of the 40 parameters, the variances have the same values, while, for the remaining 22, at least two variances are different. This latter takes place for the *LocalJitter* for all the eight vowels and syllables, for the *LocalShimmer* in seven cases, and for the *stdevF0* in seven cases.
  - *independence*: this implies two hypotheses: the first is that the observations in each class are independent of the observations in every other class, and the second is that the observations within each class are obtained through random sampling. In our case, this independence assumption is not violated due to the way the dataset has been created: each item in the dataset makes reference to a different subject, no participating subject is present twice in the dataset, and each of them has been assigned to one and only one class.
- *there is a need for non-parametric statistical analysis*: the above results forbid us to use parametric statistical analysis; rather, non-parametric statistical tests should be run; within them, one of the most frequent choices consists in the use of the Kruskal-Wallis test^52^ followed, if necessary, by the Mann-Whitney test^53^.
  - *the Kruskal-Wallis test*: with this test, we wish to check if, for any given dataset parameter under account, the medians of the values of this parameter computed for the different classes are equivalent. The results of this test, applied with a significance level of 0.05, reveal that ten parameters out of the 40 reject the above equivalence hypothesis: they are all the *stdevF0* parameters for all the vowels and syllables apart from that for the vowel /a/, and three *LocalJitter* parameters, i.e., those for the vowels /e/, /i/, and /o/. This means that, for each of them, at least one median is different from another one. This suggests that these parameters could be helpful in separating at least one class from another class. As an interesting detail, the standard deviations of F_0_ related to the vowel /u/ and to the syllables /pa/, /ta/, and /ka/ show the highest confidence in rejecting the hypothesis of equivalent medians among classes.
  - *the Mann-Whitney test*: the Kruskal-Wallis test has found the ten parameters for which at least two medians related to two classes are different, yet it cannot inform us on which these medians and these classes are. To answer this question, over each of these ten parameters, we have run the Mann-Whitney test, which can spot the classes for which the medians are different. This test performs a multiple comparison between more than two classes; therefore, the results should be adjusted through the use of a correction method. From among the available ones, in this paper, we have decided to use the Bonferroni method^54,55^, which is very frequently utilized. The results reveal that the *LocalJitter* parameters considered, i.e., those for the vowels /e/, /i/, and /o/, could separate either no pair or at most one pair of classes only so that they could not be very useful in a classification task. Each of the seven parameters related to the *stdevF0*, instead, could separate a number of class pairs ranging from one to three, so they can be more useful. Some interesting results:
    - for the *stdevF0* parameter of the /pa/ syllable, the median for the class of the *Healthy controls* is different from those of all the classes that make reference to the several degrees of the disease apart from the class containing the *Non-Dysarthric* subjects. This suggests that this parameter could be helpful in telling healthy people from those suffering from more severe levels of ALS;
    - *stdevF0* for the vowel /o/ could be useful when dividing the class with the *Moderate-Dysarthric* subjects from those containing the *Non-Dysarthric* people and the *Healthy controls*;
    - *stdevF0* for the vowel /u/ could be of help in the discrimination of the *Healthy controls* from the *Moderate-Dysarthric* and the *Mild-Dysarthric* subjects;
    - the same holds true if we consider the *stdevF0* for both the syllable /ta/ and the syllable /ka/.

- *summarizing*: from the above analysis, we can conclude that the parameters expressing the standard deviation of F_0_ for all the vowels and syllables have the highest interest from the point of view of a better division of this dataset into classes and could be used for the classification and the clustering tasks.

**Fig. 3:**
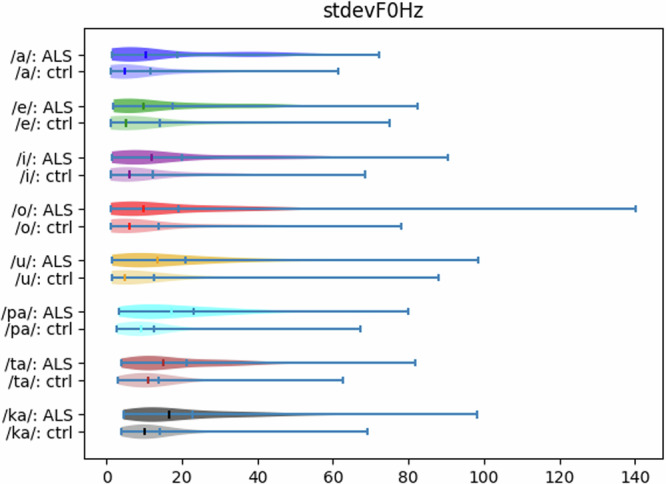
The violin plots related to the eight *stdevF0* parameters. For each parameter, we report above the people with ALS (“ALS”) and below the *Healthy control* subjects (“control”).

Figure 3 shows the violin plots for these eight parameters by considering separately the *people withALS* and the *Healthy control* subjects. For the sake of comparison, for each parameter, the violin plots of controls and ALS patients are paired. A violin plot^56^ is an extension to the classical box plot: it shows at the same time the same pieces of information as a box-and-whisker plot and the probability density of the data at different values, usually smoothed by a kernel density estimator; hence, violin plots are very informative.

By comparing the violin plots, we can notice that, for any considered parameter, both the numerical values and the shape differ between the ALS subjects and the *Healthy control* ones. Also, it can be seen that the variation ranges for these parameters are very different between the two groups: those for the people with disease are much larger than those for the *Healthy controls*. This visually confirms that the standard deviations of F_0_ for all the vowels and syllables considered could be helpful in separating the two groups.

**Fig. 4:**
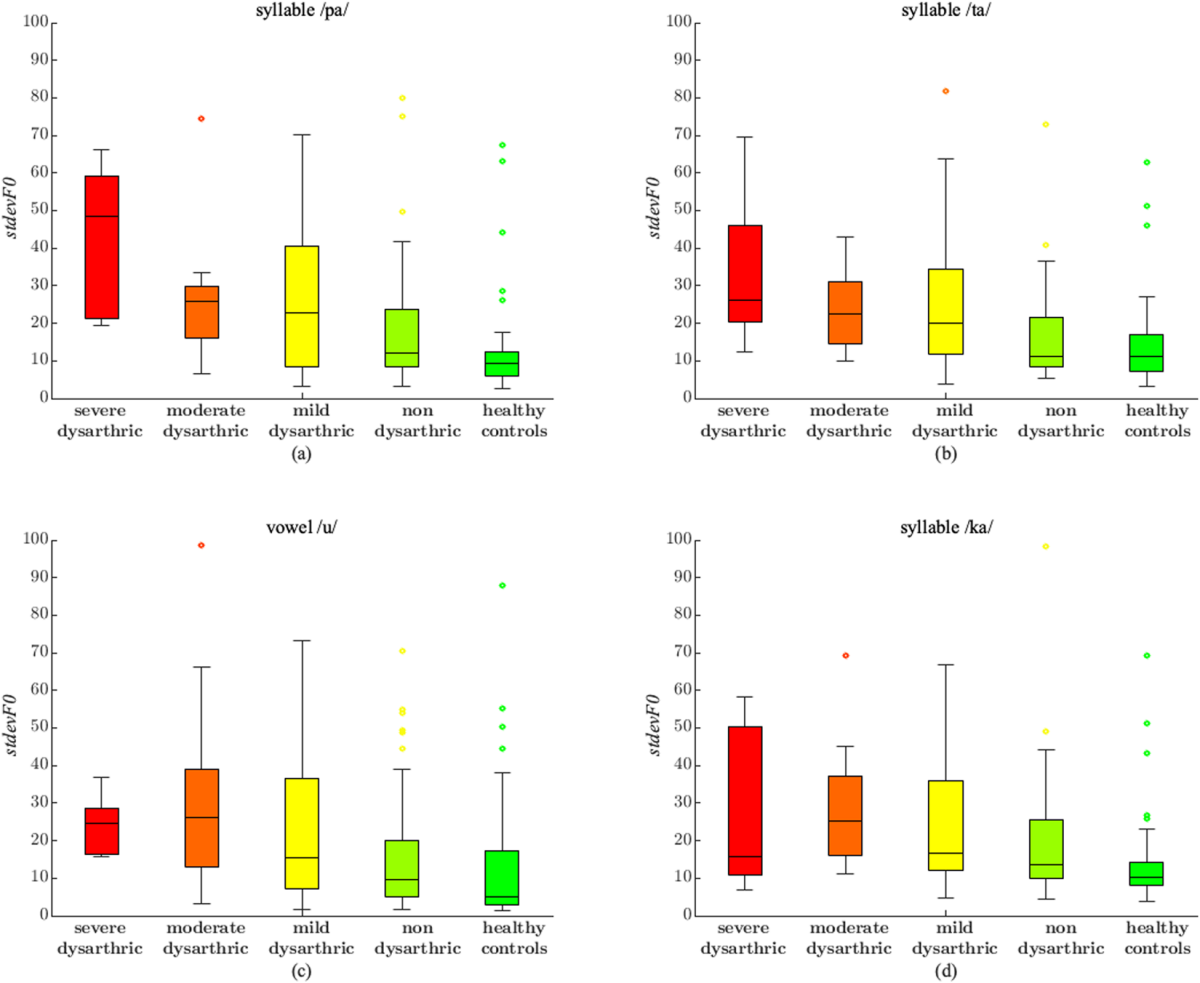
The box plots for the four most discriminating parameters. First row left pane (**a**): *stdevF0* for the syllable /pa/. First row right pane (**b**): *stdevF0* for the syllable /ta/. Second row left pane (**c**): *stdevF0* for the vowel /u/. Second row right pane (**d**): *stdevF0* for the syllable /ka/.

**Fig. 5:**
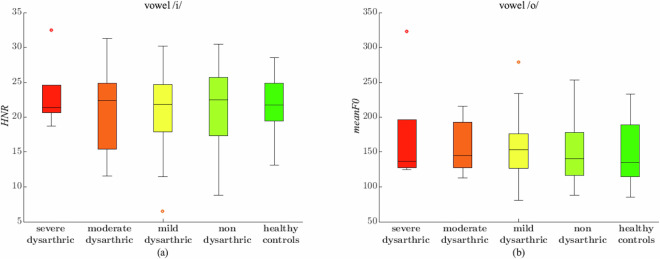
The box plots for the two least discriminating parameters. Left pane (**a**): *HNR* for the vowel /i/. Right pane (**b**): *meanF0* for the vowel /o/.

Figures 4 and 5 aim to visually compare the different relationships between the medians for different dataset parameters. Namely, in Fig. 4, we show the boxplots of the four parameters for which the Kruskal-Wallis test rejects the equivalence hypothesis with the highest confidence, i.e., in decreasing order of rejection, the *stdevF0* for /pa, /ta/, /u/, and /ka/; the panes for these four parameters show that the medians between the classes are, in many cases, different, which suggests that the corresponding classes could be divided through the use of these parameters. On the other hand, we have also taken into account the two parameters for which the Kruskal-Wallis test cannot reject the equivalence hypothesis with the highest confidence; they are, in decreasing order, the *HNR* for the vowel /i/ and the *meanF0* for the vowel /o/; the boxplots for these two parameters are depicted in the two panes of Fig. 5 and evidence that their median values for the different classes are very close to one another, which suggests that it would be hard to tell the different classes by using these variables.

### Questionnaire Analysis

For this section, we refer to the data contained in column 62 of the .xlsx file. As expected, healthy controls scored lower than ALS patients, with a median(IQR) equal to 12 (16) versus 20 (47.50). Interestingly, patients who showed dysarthric symptoms also showed a higher score on the self-report questionnaire, implying that the severity of dysarthria was directly related to the discomfort perceived by the patient (Table 3).

### Study Limitations and Outlook

The integration of appropriately selected acoustic features with digital technology is an area of research with the potential to improve diagnosis and monitoring of patients with ALS. We believe our public dataset will engage other researchers in this critical field of study to develop new technologies for clinical decision support and remote assessment. Indeed, the additional advantage of remote devices is their ability to offer patients greater access to healthcare with fewer in-person clinic visits coupled with increased frequency of data collection. In this context, VOC-ALS^17^ would pave the way for the implementation of speech assessment technologies to be used into clinical practice and to test their validity against existing assessment measures commonly used in ALS, such as perceptual rating scales and physical neurological examination.

However, an important issue that should be underlined here is that this dataset has been obtained from a group of Italian-speaking people. How the parameters measured and contained in this dataset, as well as the obtained results, will translate to other populations is not clear. In fact, the collection of this dataset has relied on the pronunciation of the five vowels and of the three syllables /pa/, /ta/, and /ka/; variations in the pronunciation of these eight phonemes in different languages could negatively impact the utility of this database and the generality of the related results presented here. This issue is general and should be considered whenever a dataset similar to ours is introduced in the literature. Probably, general guidelines could be introduced within the scientific community when introducing new datasets similar to ours. Also, transnational cooperative ways to create broader datasets could be established and followed. In the specific case of this first Italian-based dataset, a joint effort from as many countries as possible could possibly help increase it and create a much more comprehensive dataset that could be more representative of the several speakers worldwide. Furthermore, a future study including a larger sample of patients belonging to each dysarthria severity class and recruited from different countries worldwide would be desirable to identify specific features through detailed speech analysis in different languages.

In addition, this database paves the way for the future development of a longitudinal dataset in which speech recordings are performed at multiple time points in order to develop useful biomarkers for detecting subclinical abnormalities of bulbar functions, predicting the onset of dysarthria and its worsening over time.

Lastly, the inclusion of a neurological control population with dysarthria (i.e., Parkinson’s disease, dementia, stroke) as well as considering new acoustic features (e.g., speech pause analysis and speaking/articulatory rate) would be desirable in the future to gain insight into the sensitivity and specificity of our speech analysis.

In conclusion, this database currently represents the most complete and free downloadable dataset of voice signals recorded in ALS patients recruited during routine outpatient visits and deeply phenotyped. Thus, we believe that VOC-ALS represents a useful clinical dataset for developing new and more sensitive tools to efficiently assess dysarthria severity.

## Data Availability

Data processing and analysis have been performed in Matlab and Python. Dedicated scripts have been developed by us, both for processing and for analyzing data. We reiterate here that the multiple acoustic features were computed using the Parselmouth^37^, a Python library for the Praat software^38^; instead, all analysis included in the **Technical Validation** section has been performed in Matlab and in Python by using classic available functions, for example, shapiro_wilk, vartestn, kruskalwallis, multcompare, violinplot, and boxchart. The developed Python script used to extract all acoustic features from the .wav file is freely available on GitHub at https://github.com/giovannasannino/VOC-ALS.git.
